# Supplementary material for: Topological and Structural Plasticity of the Single Ig Fold and the Double Ig Fold Present in CD19
Source: Biomolecules. 2021 Aug 30;11(9):1290. doi: 10.3390/biom11091290 (PMC8470474; doi:10.3390/biom11091290)
Supplement: Supplementary file 1 [file biomolecules-11-01290-s001.zip › biomolecules-1284259-supplementary.pdf]

Supplement

Topological and Structural Plasticity of the single Ig fold and the double Ig fold present in CD19.

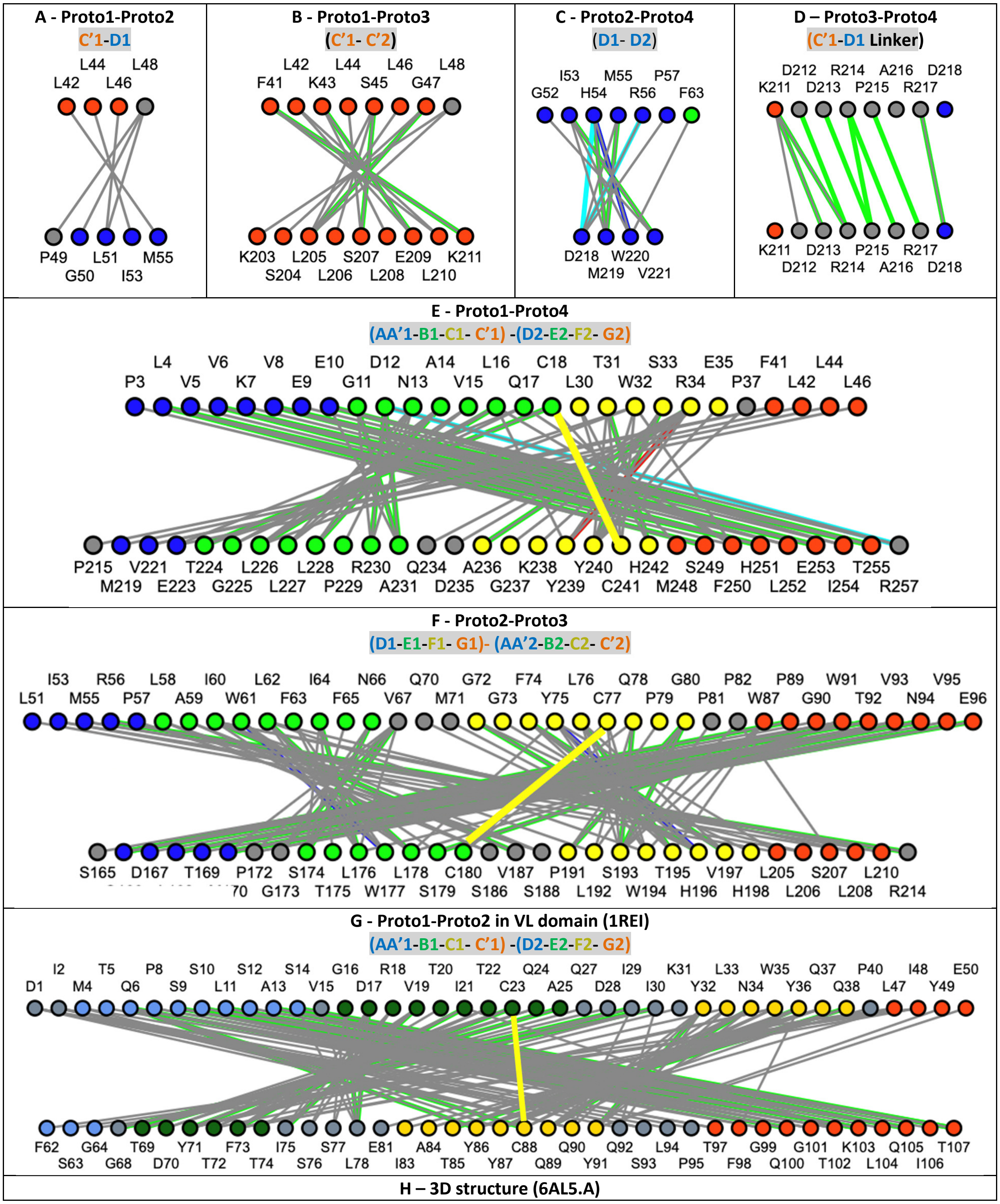

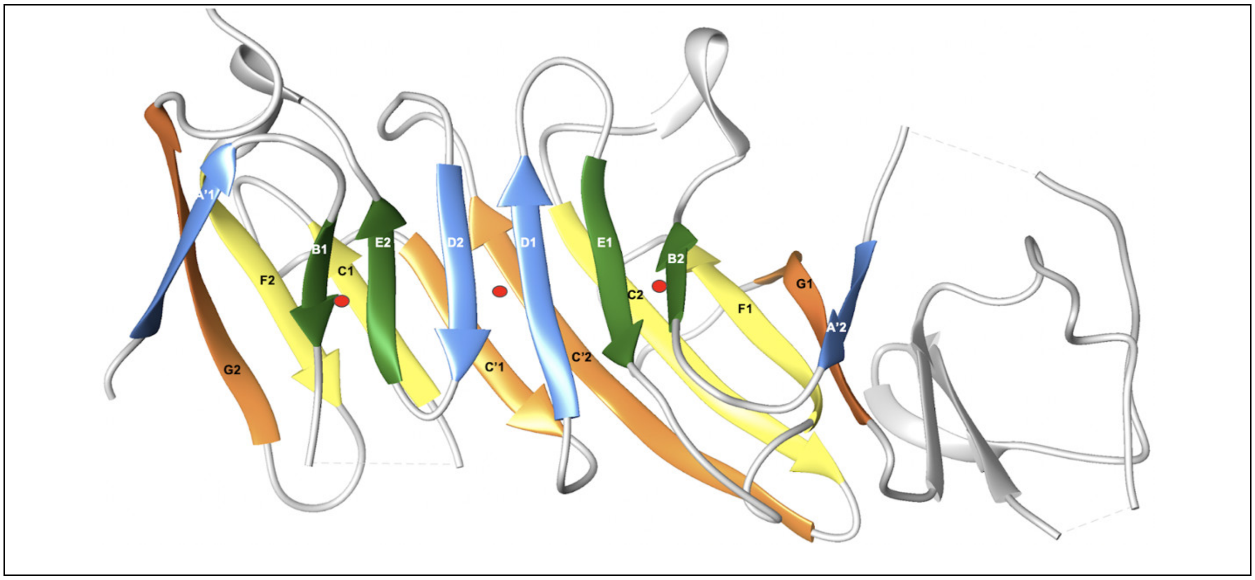

Figure S1 – CD19 double Ig domain protodomains interactions. A) Protodomains 1–2 interactions involves consecutive strands **C'1-D1** of CD19 (PDBid 6AL5 chain A) that are separated by a short linker loop between the two sheets. Unlike a regular Ig domain, they run in opposite directions **B) Protodomains 1–3 interactions** involves strands **C'1- C'2** on the same sheet at the interface between the Ig domain formed by protodomains 1-4 and 2-3 in E) and F)**. C) Protodomains 2 –4 interactions** involve strands **D1-D2. D) Protodomains 3–4 interactions** do not involve direct strand-strand residue contacts. However, the **C'2-D2 linker** (residues K211-D218) between the two protodomains forms a highly connected (H-bonded) substructure connecting the two strands across beta sheets. **E) Protodomains 1-4 interactions** show a well-formed regular Ig domain. **F) Protodomains 2-3 interactions** show an inverse pattern of a regular Ig-domain, since protodomain 2 and 3 and in a reverse order in sequence vs. a regular Ig domain. **G) Protodomains 1–2 interactions** in a regular IgV domain (VL domain of 1REI) for reference. Colors: VdW interactions are shown in grey; canonical Cys bridges are shown in yellow; H-bonds in green; charge-charge in cyan, aromatic-aromatic in blue, charged-aromatic in red. **H) CD19 ribbon structure** showing axes of symmetry perpendicular to the paper plane. See Figure 4 and main text for more details. Links to interactive 3D visualization of interactions for CD19 (PDBid: 6AL5): (https://structure.ncbi.nlm.nih.gov/icn3d/share.html?braxhyx8kzdtqtNU6 accessed 27 Aug 2021, and for Bence Jones protein interactions (PDBid: 1REI): https://structure.ncbi.nlm.nih.gov/icn3d/share.html?x3VMo23SdZDKniys5 accessed 27 Aug 2021).

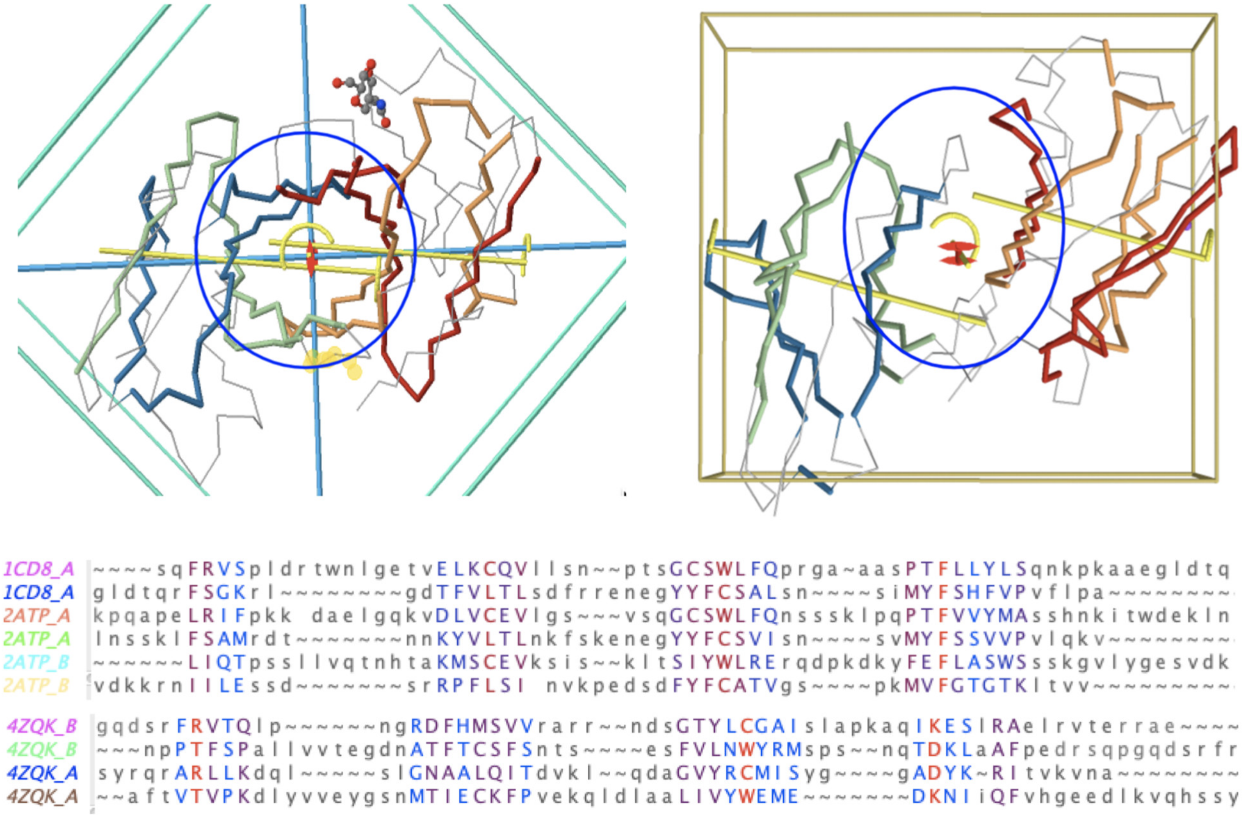

Figure S2 - Comparing quaternary pseudo-symmetry of CD8 and PD1-PD-L1. Top Left) CD8a/CD8b interface (2ATP) forms a canonical VH-VL interface, keeping a quasi-colinearity between domain level C2 axes of symmetry, keeping therefore a quasi-D2 overall symmetry in terms of protodomains (C2 axes of symmetry in yellow). **Top Right) PD-1/PD-L1 interface** (4ZQK) conserving a quaternary C2 symmetry but with a shift of the internal axes of symmetry of Ig domains of PD-1 and PD-L1. Tertiary and quaternary symmetries as detected by the program CEsymm. CD8ab heterodimer (2ATP) aligns with PD-1/PD-L1 receptor-ligand pair within 3.83 A RMSD over 178 residues with 16% sequence identity (https://www.ncbi.nlm.nih.gov/Structure/icn3d/full.html?showalignseq=1&align=2atp,4zqk&atype=0 accessed 27 Aug 2021). **Bottom) Optimized protodomains alignments** CD8aa (human) (PDBid: 1CD8) and CD8ab (mouse) (PDBid: 2ATP) RMSD relative to the first protodomain: 1.61 A, 0.52 A, 1.95 A, 0.89 A, 1.54 A ; PD-L1/PD-1 (human) 4ZQK RMSD relative to the first protodomain: RMSD 1.73 A, 1.53 A, 1.84 A.

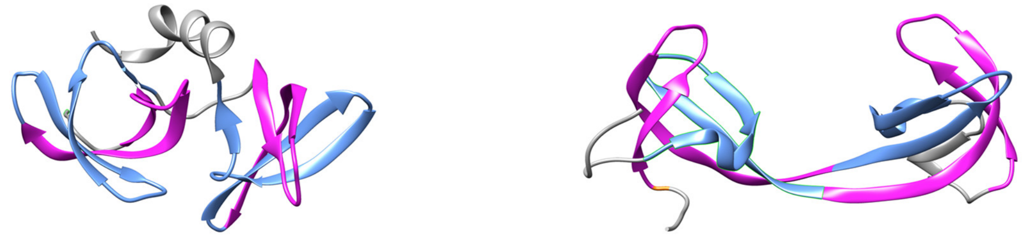

A - Tandem Tudor
protodomains 1/3 blue – 2/4 magenta
sequence domain 1 (p1-p2) green – domain 2 (p3-p4). Orange
structural domain 1 p1-p2 ; domain 2 p3-p4

B - Double Tudor. (interdigitated)
protodomains 1/3 blue – 2/4 magenta
sequence domain 1 (p1-p2) green – domain 2 (p3-p4). orange
structural domain 1 p1-p4 ; domain 2 p2-p3

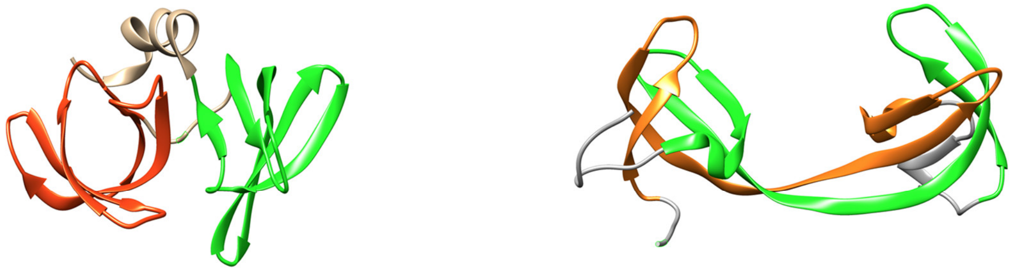

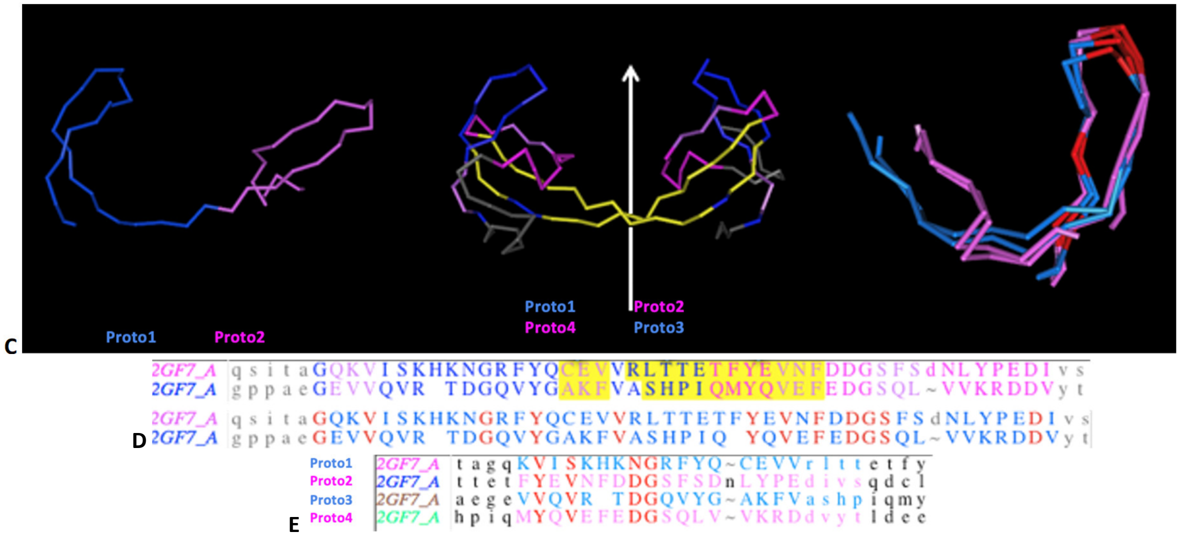

Figure S3 - Comparing Tandem Tudor Domains to the interdigitated double Tudor. A) Tandem Tudor (53BP1 PDBids: 1XNI/4X34) domain 1 green; domain 2 orange protodomains p1/p3 blue p3/p4 magenta. **B) Double Tudor** (JMJD2A: PDBid 2GF7) same coloring. **C) Pseudo symmetry of the interdigitated double tudor domain: left** domain 1 with sequential protodomains 1+2 in blue and magenta (3+4 similar); **center:** pseudo symmetric assembly forming 2 structural domains p1+p4 and p2+p3; **right:** superimposed protodomains (red = conserved residues). **D) Tudor domain alignment in sequence** (p1-p2) vs. p3-p4 or interdigitated double Tudor. **E) Protodomains alignment** p1-p2-p3-p4

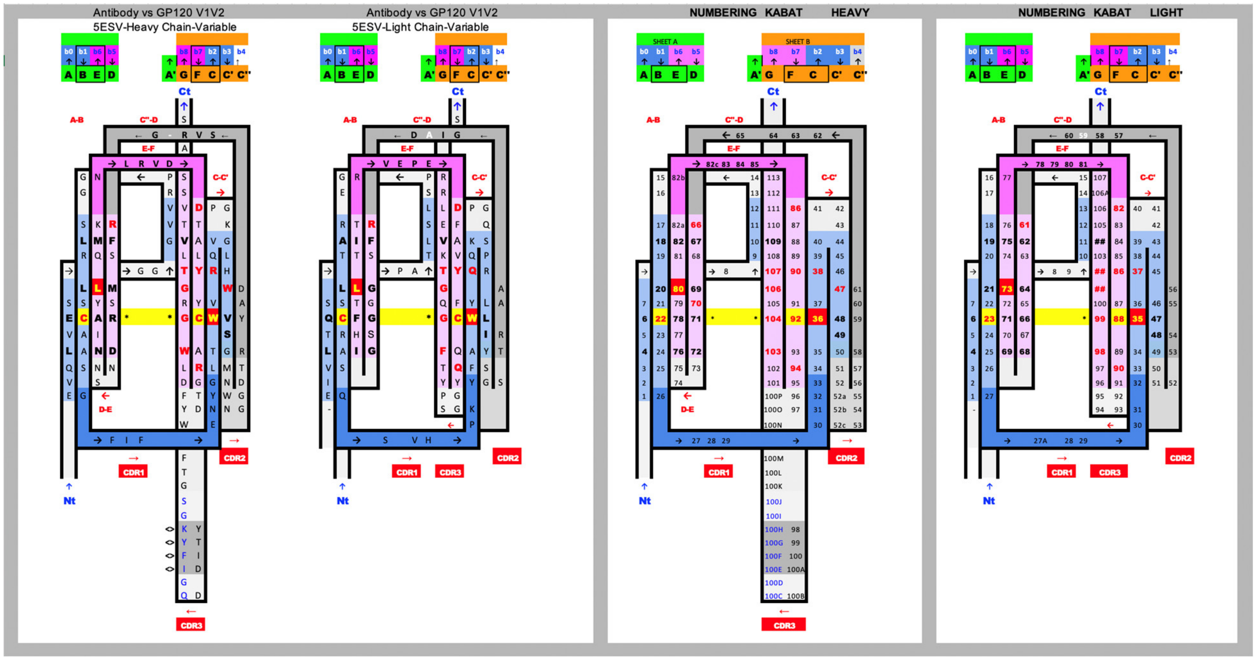

Figure S4 – Topology-Sequence Maps of a IgVH and IgVL domains with corresponding Kabat numbering

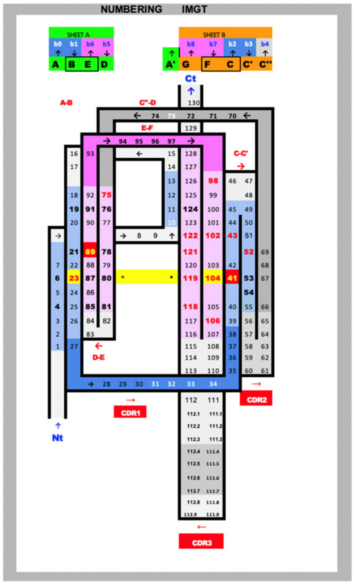

Figure S5 – Topology-Sequence map of IgV domains with IMGT numbering
